# Design and Synthesis of New Quinoxaline Analogs to Regenerate Sensory Auditory Hair Cells

**DOI:** 10.3390/cells14241946

**Published:** 2025-12-08

**Authors:** Sonia M. Rocha-Sanchez, Elton Jeffrey North, Lilian E. Calisto, Brock M. Barthol, Kenneth D. Nguyen, Jigar P. Sethiya

**Affiliations:** 1 Department of Oral Biology, School of Dentistry, Creighton University, Omaha, NE 68178, USA; liliancalisto@creighton.edu (L.E.C.); brockbarthol@creighton.edu (B.M.B.); kennethnguyen@creighton.edu (K.D.N.); 2 Department of Pharmacy Sciences, School of Pharmacy and Health Professions, Creighton University, Omaha, NE 68178, USA; jeffreynorth@creighton.edu (E.J.N.); jigarsethiya@creighton.edu (J.P.S.)

**Keywords:** mammalian, auditory hair cells, supporting cells, regeneration, proliferation, mitotic division, hearing loss, zebrafish

## Abstract

**Highlights:**

**What are the main findings?**

- Two new lead quinoxaline derivatives promote measurable auditory supporting cell proliferation and the generation of new sensory hair cells in zebrafish neuromasts and mouse organs of Corti without inducing apoptosis or impacting the function of the sensory hair cells.
- Improved hearing in mice at certain treatment doses, along with observed proliferation patterns, indicates compound-specific effects and suggests the engagement of canonical pro-growth pathways, including Wnt/β-catenin and MAP3K1-IKK-NF-κB.

**What are the implications of the main finding?**

- Enhancing the proliferation of auditory supporting cells through pharmacological modulation could improve the mitotic regenerative capacity of mammalian sensory epithelia, ultimately creating more favorable conditions for future strategies aimed at regenerating auditory hair cells.
- These findings highlight two quinoxaline derivatives as promising candidates for further exploration into their role in treatment pathways, long-term safety, and potential use in regeneration-based strategies for sensorineural hearing loss.

**Abstract:**

No pharmacological interventions exist that can restore or preserve auditory function in the mammalian cochlea. Auditory hair cells (HCs) do not spontaneously regenerate, leading to permanent hearing loss. In non-mammalian vertebrates, HC regeneration happens through proliferation and differentiation of their clonally related supporting cells (SCs). The present study supports the potential of quinoxaline (Qx), a nonsteroidal anti-inflammatory compound, to stimulate SC proliferation in the auditory sensory epithelium, a process that may prime the tissue for future HC regeneration. We synthesized a series of Qx derivatives by introducing various substitutions, ranging from hydrophilic to lipophilic. Seventy analogs were generated and tested in vitro and in vivo. Among those, only one (Qx-100) exhibited the best medicinal chemistry profile and was further modified to expand the structure–activity relationship of the chemotype, develop additional analogs, and optimize potency, bioavailability, and in vivo efficacy. Ten new lead variants were generated. Of those, Qx-294 and Qx-301 demonstrated promising in vitro Absorption, Distribution, Metabolism, and Excretion (ADME) profiles and were selected for further testing. Overall, both compounds were rapidly absorbed in zebrafish and mice and promoted cell proliferation in vitro and in vivo without signs of apoptosis, supporting their potential for sensory HC regeneration.

## 1. Introduction

Sensorineural hearing loss caused by the irreversible loss of cochlear hair cells (HCs) is a widespread deficit affecting an estimated 5% of the global population [1]. Sensory HCs, specialized mechanosensory receptors in the organ of Corti (OC), convert sound-induced mechanical vibrations into neural signals. In mammals, once destroyed by noise exposure, ototoxic drugs, aging, or genetic factors, these sensory HCs do not regenerate, leading to permanent auditory dysfunction [2]. In contrast, non-mammalian vertebrates, such as birds and fish, exhibit a remarkable capacity for spontaneous HC regeneration through the proliferation of supporting cells (SCs) or the direct transdifferentiation of those cells into new HCs [3,4]. The stark contrast in regenerative ability between mammals and non-mammals has motivated extensive research into the molecular pathways that regulate HC development and regeneration. Key signaling pathways—Notch, Wnt/β-catenin, FGF, and Hippo—have emerged as central regulators supporting cell fate and proliferation [5,6,7]. Furthermore, transcription factors including ATOH1, GFI1, and POU4F3 play pivotal roles in HC differentiation and have been shown to partially induce HC-like phenotypes when ectopically expressed in SCs [8,9,10,11]. Likewise, recent innovations in gene therapy, CRISPR/Cas9 genome editing, and stem cell-derived organoid models provide promising platforms for manipulating these pathways and studying the regenerative potential in human systems [12,13,14]. Despite these advances, translating regenerative strategies into clinically viable therapies remains a significant challenge, particularly due to limited cell plasticity and epigenetic constraints in the adult mammalian cochlea [11]. Previous work by our group has highlighted the role of cell cycle regulation in the mammalian inner ear, particularly the contribution of the retinoblastoma family of pocket proteins and their upstream regulator, Cyclin D1, in auditory HC and SC proliferation [15,16,17,18]. Building on that work and its insights into how manipulating cell cycle regulators can influence cell proliferation and differentiation in the cochlea, we identified quinoxaline (Qx; C_8_H_6_N_2_), a heterocyclic compound that displays otoprotective and proliferative effects in the OC [19]. Qx represents a compelling core in medicinal chemistry. From a structural perspective, it is a fused benzene and 1,4-azine ring system, and is a bioisostere of quinazoline, indole, benzimidazole, benzothiophene, and benzothiazole. Qx-containing compounds can bind to various targets, which makes them privileged structures. Examples of marketed drugs bearing a quinoxaline structure include the antileprotic clofazimine, the smoking cessation aid varenicline, the antitumor antibiotic phenazinomycin, the antitumor erdafitinib (an inhibitor of the fibroblast growth factor receptor, FGFR), and the α-adrenergic agonist abrimonidine [20]. In its original structure, Qx has demonstrated some antiapoptotic properties [21] and protective effects against the harmful effects of cisplatin and gentamicin, as well as partial protection against neomycin, without impacting the mechanotransduction activity of HCs [19].

The therapeutically induced generation of new sensory HCs has been considered for many years. Nevertheless, no pharmacological alternatives are yet available to safely stimulate HC regeneration through controlled SC proliferation. The present study focused on applying medicinal chemistry to enhance Qx’s preclinical properties, thereby expanding the structure–activity relationship of the generated chemotypes, identifying proliferative analogs, and optimizing potency, bioavailability, and in vivo efficacy. Two lead variants, Qx-294 and Qx-301, have been identified as part of this effort, demonstrating promising in vitro Absorption, Distribution, Metabolism, and Excretion (ADME) profiles. Both compounds have been shown to exhibit rapid absorption in zebrafish and induce SC proliferation in vitro (using HEI-OC1 cells and cochlear explants) and in vivo (in zebrafish neuromasts and mouse inner ears), without signs of apoptosis. Overall, the results support the potential of these two compounds in further studies examining cochlear HC regeneration.

## 2. Materials and Methods

### 2.1. General Chemistry Methods and Instrumentation

All the reagents, glassware, solvents, and chemicals were purchased from commercially available sources. All the chemicals were reagent-grade and were used directly without further purification. The reactions were tracked and monitored by using fluorescent-silica gel-coated thin layer chromatography (TLC) plates, and the spots were visualized using a UV lamp. The purification of the compounds was performed using flash chromatography on a Biotage^®^ Isolera One with a Biotage^®^ silica gel column (Uppsala, Sweden). ^1^H NMR were recorded on a 400 MHz Bruker NMR system (Billerica, MA, USA), and the chemical shifts were reported relative to the solvent peaks. Mass spectra were obtained on an Agilent 1200/AB Sciex API 5500 QTrap LC/MS/MS system (Marlborough, MA, USA) using electrospray ionization and a single quadrupole analyzer (Q1).

### 2.2. General Synthetic Method for Straight-Chain 2-Aminoquinoxaline Analogs

2-aminoquinoxaline (0.689 mmol) was reacted with sodium hydride (1.032 mmol, 60% dispersed in mineral oil) in tetrahydrofuran in a round-bottom flask on a dry ice bath for five minutes. Iodoalkane (1.032 mmol) was added to the reaction and stirred for 24 h at room temperature. Cold water was added to quench the reaction mixture, followed by extraction with ethyl acetate and brine (2×). The organic layer was dried over sodium sulfate, filtered, and evaporated under reduced pressure. The crude residue was purified using flash chromatography with silica gel stationary phase and ethyl acetate in hexane gradient for mobile phase.

2-(ethylamine)-quinoxaline (292): 7.6 mg (7.2%); ^1^H NMR (CDCl_3_) δ = 1.41 (t, J = 8 Hz, 3H), 3.64 (q, J = 8 Hz, 2H), 7.46 (t, J = 8 Hz, 1H), 7.50 (t, J = 8 Hz, 1H), 7.75 (d, J = 8 Hz, 1H), 7.93 (d, J = 8 Hz, 1H), 8.40 (s, 1H). ESI-MS calculated for C_10_H_12_N_3_: 174.2, found: 175.0 [M+H]^+^.
2-(propylamine)-quinoxaline (293): 12.0 mg (10.5%); ^1^H NMR (CDCl_3_) δ = 0.97 (t, J = 8 Hz, 3H), 1.65 (sex, J = 8 Hz, 2H), 3.44 (q, J = 8 Hz, 2H), 7.30 (t, J = 8 Hz, 1H), 7.49 (t, J = 8 Hz, 1H), 7.61 (d, J = 8 Hz, 1H), 7.78 (d, J = 8 Hz, 1H), 8.15 (s, 1H). ESI-MS calculated for C_11_H_14_N_3_: 188.3, found: 188.9 [M+H]^+^.

### 2.3. General Synthetic Method for Cyclic 2-Aminoquinoxaline Analogs

2-chloroquinoxaline (0.608 mmol), potassium carbonate (1.215 mmol), and amine (3.038 mmol) were dissolved in 2.4 mL of dioxane/water (9:1) and heated at 105 °C in a sealed tube for 3 h The crude mixture was filtered, and the solid was then dissolved in ethyl acetate and washed twice with brine. The organic layer was dried over sodium sulfate, filtered, and evaporated under reduced pressure. The crude residue was purified using flash chromatography with a silica gel stationary phase and an ethyl acetate in hexane gradient as the mobile phase.

2-(morpholine)-quinoxaline (294): 13.6 mg (10.4%); ^1^H NMR (CDCl_3_) δ = 3.79–3.81 (m, 4H), 3.89–3.91 (m, 4H), 7.45 (t, J = 8 Hz, 1H), 7.62 (t, J = 8 Hz, 1H), 7.74 (d, J = 8 Hz, 1H), 7.91 (d, J = 8 Hz, 1H), 8.60 (s, 1H). ESI-MS calculated for C_12_H_14_N_3_O: 216.3, found: 216.9 [M+H]^+^.
2-(thiomorpholine)-quinoxaline (295): 11.0 mg (7.8%); ^1^H NMR (CDCl_3_) δ = 2.77–2.80 (m, 4H), 4.17–4.20 (m, 4H), 7.43 (t, J = 8 Hz, 1H), 7.60 (t, J = 8 Hz, 1H), 7.72 (d, J = 8 Hz, 1H), 7.90 (d, J = 8 Hz, 1H), 8.56 (s, 1H). ESI-MS calculated for C_12_H_14_N_3_S: 232.3, found: 233.0 [M+H]^+^.
2-(4-acetylpiperazine)-quinoxaline (296): 14.8 mg (9.5%); ^1^H NMR (CDCl_3_) δ = 2.19 (s, 3H), 3.66–3.69 (m, 2H), 3.78–3.85 (m, 4H), 3.85–3.90 (m, 2H), 7.46 (t, J = 8 Hz, 1H), 7.63 (t, J = 8 Hz, 1H), 7.73 (d, J = 8 Hz, 1H), 7.92 (d, J = 8 Hz, 1H), 8.62 (s, 1H). ESI-MS calculated for C_14_H_17_N_4_O: 257.3, found: 258.0 [M+H]^+^.
2-(piperazine)-quinoxaline (297): 12.3 mg (9.4%); ^1^H NMR (CDCl_3_) δ = 2.95 (s, 2H), 3.07–3.09 (m, 4H), 3.80–3.83 (m, 4H), 7.42 (t, J = 8 Hz, 1H), 7.60 (t, J = 8 Hz, 1H), 7.69 (d, J = 8 Hz, 1H), 7.89 (d, J = 8 Hz, 1H), 8.60 (s, 1H). ESI-MS calculated for C_12_H_15_N_4_: 215.3, found: 216.0 [M+H]^+^.
2-(4-methylpiperazine)-quinoxaline (298): 10.5 mg (7.6%); ^1^H NMR (CDCl_3_) δ = 2.42 (s, 3H), 2.62–2.65 (m, 4H), 3.85–3.88 (m, 4H), 7.42 (t, J = 8 Hz, 1H), 7.60 (t, J = 8 Hz, 1H), 7.70 (d, J = 8 Hz, 1H), 7.89 (d, J = 8 Hz, 1H), 8.60 (s, 1H). ESI-MS calculated for C_13_H_17_N_4_: 229.3, found: 230.0 [M+H]^+^.
2-(4-ethylpiperazine)-quinoxaline (299): 11.9 mg (8.1%); ^1^H NMR (CDCl_3_) δ = 1.18 (t, J = 8 Hz, 3H), 2.53 (q, J = 8 Hz, 2H), 2.64–2.67 (m, 4H), 3.85–3.88 (m, 4H), 7.41 (t, J = 8 Hz, 1H), 7.59 (t, J = 8 Hz, 1H), 7.69 (d, J = 8 Hz, 1H), 7.88 (d, J = 8 Hz, 1H), 8.60 (s, 1H). ESI-MS calculated for C_14_H_19_N_4_: 243.3, found: 243.9 [M+H]^+^.
2-(piperidine)-quinoxaline (300): 24.2 mg (18.7%); ^1^H NMR (CDCl_3_) δ = 1.74 (br, 6H), 3.80 (br, 4H), 7.39 (t, J = 8 Hz, 1H), 7.58 (t, J = 8 Hz, 1H), 7,71 (d, J = 8 Hz, 1H), 7.87 (d, J = 8 Hz, 1H), 8.61 (s, 1H). ESI-MS calculated for C_13_H_16_N_3_: 214.3, found: 214.9 [M+H]^+^.
2-(pyrrolidine)-quinoxaline (301): 12.5 mg (10.3%); ^1^H NMR (CDCl_3_) δ = 2.08–2.12 (m, 4H), 3.69–3.72 (m, 4H), 7.36 (t, J = 8 Hz, 1H), 7.58 (t, J = 8 Hz, 1H), 7.72 (d, J = 8 Hz, 1H), 7.88 (d, J = 8 Hz, 1H), 8.37 (s, 1H). ESI-MS calculated for C_12_H_14_N_3_: 200.3, found: 200.7 [M+H]^+^.

### 2.4. Cell Culture

HEI-OC1 cells derived from the mouse OC (kindly provided by Dr. F. Kalinec) were cultured under permissive conditions (33 °C, 10% CO_2_) as described in [22]. Additionally, we assessed the activity of Qx-derived compounds in the mouse neuroblastoma cell line, Neuro-2A, and the human fibrosarcoma HT-1080 (ATCC, Manassas, VA, USA) following the standard cell culture guidelines provided by the vendor.

### 2.5. Animals

Experiments with mice (*Mus musculus*) and zebrafish (*Danio rerio*) were conducted in accordance with the National Institutes of Health Guide for the Care and Use of Laboratory Animals. Zebrafish experimental larvae of either sex were obtained by pair matings of adult fish maintained at the Creighton University Animal Research Facility. The study protocol was approved by the Creighton University Institutional Animal Care and Use Committee (IACUC # 1131). Information on the allocation of experimental animals is provided in Table 1.

### 2.6. Zebrafish

The GFP (*Tg(brn3c: GFP*) zebrafish larvae expressing HC membrane-bound GFP [19] were used to evaluate the effect of Qx-294 and Qx-301 on HCs and SCs of the lateral line neuromasts. Healthy 5-dpf zebrafish, maintained at 28.5 °C, were randomly distributed into Petri dishes at a density of 50 embryos/larvae per 100 mm^2^ and exposed to various concentrations of Qx-294 and Qx-301 (50, 100, 150, 200, 250, and 300 μg/mL) diluted in E3 (5 mM NaCl, 0.17 mM KCl, 0.33 mM CaCl2 and 0.33 MgSO4, pH 7.2) medium for 24 h. Following treatment, the animals were transferred to E3 media for 30 min to recover, then cryoanesthetized and fixed with 4% paraformaldehyde (PFA) overnight at 4 °C. The following neuromasts were analyzed in our studies: IO4 (infraorbital 4), OP1 (opercular 1), M2 (mandibular 2), O1 (otic 1), O2, and MI2 (middle 2). An arbitrary scoring protocol was used to rank the morphology of each neuromast after treatment as follows: 1 (normal rosette-like shape, HCs look normal, HC bundles are properly arranged), 2 (normal rosette-like shape, a few HCs appear to be missing, but HC bundles are properly arranged), 3 (normal rosette-like morphology but several HCs are gone and HC bundles look disturbed), 4 (the rosette-like shape is lost in certain areas, few HCs, and few disrupted HC bundles), and 5 (no rosette-like shape, one or two dispersed HCs and no HC bundles). At least 25 animals per treatment were assessed, for a total of 200 across the entire study. The assessment was performed in a double-blind and unbiased manner. Scores were expressed as percentages of the total of neuromasts analyzed per treatment.

### 2.7. Mice

C57BL/6J male and female mice between postnatal day 24 and 30 P-24-30) were randomized into control and experimental groups (9 mice per group per compound), with each experimental group receiving a single intraperitoneal (IP) injection of Qx-294 or Qx-301 at a dose of 300 mg/kg body weight, dissolved in dimethyl sulfoxide (DMSO). Controls consisted of treatment with DMSO alone (vehicle). A total of 90 adult wild-type C57/BL6 mice were used in this study. Based on the analysis, animals were examined at various times following treatment as described below.

### 2.8. Immunolabeling

Cochlear dissection in mice and immunofluorescence experiments on zebrafish neuromasts and mouse cochleae were performed as previously described [15,16,17,18,19,23]. The antibody and labeling kits used in this study included Otoferlin (Developmental Studies Hybridoma Bank, Iowa City, IA, USA, HCS-1), GFP (Novus Biologicals, NB100-1614), and rabbit anti-myosin VIIa (M7a) (Thermo Scientific, Waltham, MA, USA, PA1-936). Species-specific Alexa Fluor-conjugated secondary antibodies were used for detection. Phalloidin conjugated to Alexa 488 (Thermo Scientific, Waltham, MA, USA, O7466) and DAPI were used for labeling stereocilia and the nucleus, respectively.

### 2.9. Proliferation and Apoptosis Assays

**Figure 1:**
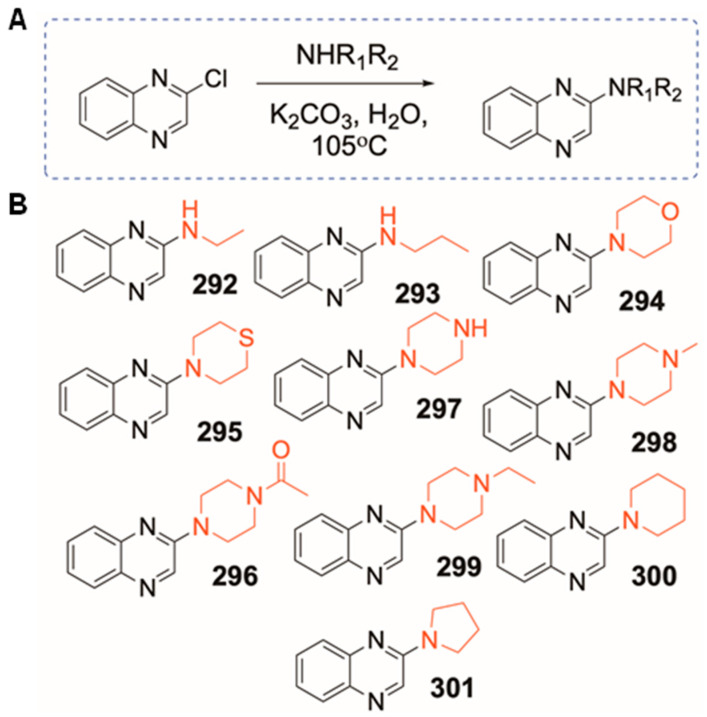
Synthetic Pathway for generating Qx analogs. (**A**) Percentage yield Qx-294: 10.4%; Qx-301: 10.3%. (**B**) Analogs were characterized using ^1^H NMR, ^13^C NMR, mass spectrometry, and HPLC. Modifications to the Qx structure are shown in red.

In vitro cell proliferation effects of all ten initial compounds (Figure 1A,B) were tested on HEI-OC1, Neuro-2A and HT-1080 cells by TACS^®^ 3-(4,5-dimethylthiazol-2-yl)-2,5-diphenyltetrazolium bromide (MTT) Cell Proliferation Assay (R&D Systems, Minneapolis, MN, USA, 4890-025-K), following the manufacturer’s protocol. Absorbance was measured with SpectraMax^®^ M Series Multi-Mode Microplate Reader with Soft Max Pro 7.2 GxP Software (Molecular Devices, Sunnyvale, CA, USA), and the average OD in control cells was taken as 100% of viability. Upon selecting the most viable compounds (Qx-294 and Qx-301), we proceeded with in vivo experiments. For zebrafish treatment, animals were incubated for varied times with different concentrations (50, 100, 150, 200, 250, and 300 μg/mL) of Qx-294 or Qx-301 diluted in swimming (E3) media or administered via IP injections. Mice treatment followed the same procedures as described in [18]. Following treatment, animals or tissue samples were incubated in Click-it EdU or Click-it Plus TUNEL kits (Thermo Scientific, Waltham, MA, USA), following the manufacturer’s instructions to label proliferating and apoptotic cells, respectively.

### 2.10. FM1-43

The styryl pyridinium dye FM1-43 provides an optical measure of mechanotransduction channel function and, therefore, was used as a proxy for the viability of the neuromast cells treated with QX-294 or QX-301, as well as indirect information on the functional capability of newly formed HCs. FM1-43FX (Thermo Scientific, Waltham, MA, USA) uptake experiments in zebrafish were performed as previously described [19]. Zebrafish were incubated with E3 alone (control) or in the presence of Qx-294 or Qx-301, both at 300 μM, for 7 h and then immediately exposed to 3 μM FM1-43FX for 40 s. Alternatively, animals were transferred to a fresh E3 solution for 1 h and then exposed to the dye. For mouse treatment, 24, 48, and 72 h after animals were injected with 300 μg/mL Qx-294 or Qx-301, their cochleae were dissected, transiently exposed to FM1-43 FX dye (1 mg/mL) for 45 s, and then fixed in 4% PFA. All animals were P28 at completion of the treatment. Fluorescence incorporated was quantified according to [19] using ImageJ (ImageJ2 1.54r).

### 2.11. Confocal Imaging

Confocal imaging was performed using either an upright Zeiss LSM 710 microscope with seven laser lines (405, 458, 488, 514, 561, 594, and 633 nm) or a Leica TCS SP8 laser scanning confocal microscope (Leica Microsystems, Wetzlar, Germany) equipped with 40X/1.3 NA oil immersion objectives and HyD hybrid detectors. Z-stack images were acquired using the 40X or 63X (NA 1.4 oil) objective at a 2× zoom, with sectioning automatically set to optimal. Images were acquired and processed using ZEN 2.3 Black Edition (Carl Zeiss Microscopy) or the Leica LAS X software (version 5.3.1). Images represent maximum-intensity projections of confocal Z-stacks from experimental animals and tissues processed in parallel. Exposure and acquisition settings were matched across groups. Only linear adjustments were made to brightness and contrast, and the final figures were assembled using Photoshop (version 27.0) and Illustrator (version 30.0) software (Adobe). Minor color differences in the experiments’ labeling resulted from multi-year data collection across different microscope platforms.

### 2.12. Auditory Function

Mice from all groups underwent auditory brainstem response (ABR) and distortion product otoacoustic emissions (DPOAEs) tests before the initiation of treatment with either compounds or vehicle, and at 24, 48, and 72 h post-treatment, as described below. After audiometric testing, pre-treatment mice were placed in a warm and clean cage and monitored until they were awake and able to remain upright in a sternal position. Alternatively, post-treatment mice had their cochleae processed for histological examination.

### 2.13. ABR

Mice were anesthetized with a ketamine (18 mg/mL) and xylazine (2 mg/mL) solution (5–9 μL per gram body weight injected intraperitoneally). Core body temperature was maintained at 37.0 ± 0.1 C using a homeothermic heating pad system (FHC, Inc., Bowdoin, ME, USA). Tone burst stimuli were generated and controlled using Tucker Davis Technologies (TDT, Gainesville, FL, USA) System III (RX6, PA5 components). Tone bursts at 8, 16, 32, and 41.2 kHz had 1.0 ms rise-fall times with 1.0 ms plateau (3 ms total duration) and alternating stimulus polarity. Stimuli for ABR testing were calibrated using a Bruel & Kjaar type 4138 ¼” microphone and Nexus type 2691 conditioning amplifier. Stimuli were calibrated in dB peSPL and presented via high-frequency transducers (TDT SA1 driver, MF1 speakers) coupled at the ear via PE tubing. Auditory stimuli were presented at a rate of 17 stimuli per second.

### 2.14. DPOAE Recording and Data Analysis

Methods for recording distortion product otoacoustic emissions (DPOAEs) were similar to those previously described [24,25,26]. Stimuli for DPOAEs were generated and controlled with modules from TDT. Pure tone frequencies (f1, f2, f2/f1 ratio ¼ 1.25), at equal levels (L1 ¼ L2 ¼ 60 dBSPL), 150 ms duration, were generated with independent sources (TDT RX6 processor) and routed through separate drivers to mix acoustically in the ear canal (via plastic tubing placed securely at the external acoustic meatus). Stimuli were calibrated in a 0.1 mL coupler, which simulates the volume of the mouse ear canal. Stimulus frequencies for the primaries are such that geometric mean (GM ¼ (f1 x f2)0.5) frequencies ranged from 6.0 to 48.5 kHz (at least eight frequencies per octave). Ear canal sound pressure levels were recorded using a low-noise probe microphone (Etymotic ER 10Bþ). The microphone output was amplified and fed into the TDT RX6 processor for digital sampling, spectral averaging, and fast Fourier transform (FFT) processing. The amplitude of f1, f2, and the cubic difference distortion product (2f1–f2) were measured from the FFT waveform. The noise floor was measured from the amplitudes in the fifth and twelfth frequency bins above and below (±60 and 120, resp.) the 2f1-f2 component. For statistical comparison (Two-Way ANOVA), the mean DPOAE amplitude across all tested primary frequency pairs was calculated and compared between treatment and control groups [25].

### 2.15. In Vitro Absorption, Distribution, Metabolism, Excretion, and Toxicity (ADMETox)

#### 2.15.1. Cytotoxicity

The cytotoxicity of Qx-294 and Qx-301 compounds was assessed in the mouse auditory HEI-OC1 cell line using the (MTT) colorimetric microculture assay method as previously described [19]. HEI-OC1 cells were cultured in DMEM (Corning, NY, USA, 10-013-CVC) supplemented with 10% fetal bovine serum (FBS) (Pansera) and 50 μg/mL ampicillin for 12 h. For cytotoxic assessment, increasing concentrations of Qx-294 and Qx-301 were added to a HEI-OC1 cell suspension (5 × 10^4^ cells/well). Cell growth was determined 72 h later by adding 50 µL of MTT (2.5 mg/mL). This was reduced by mitochondrial dehydrogenase of viable cells in an insoluble blue formazan product during the 4 h contact period at 37 °C. After the supernatant was removed, the formazan crystals were solubilized by adding DMSO (100 µL). These plates were read at 570 nm with SpectraMax^®^ M Series Multi-Mode Microplate Reader with Soft Max Pro 7.2 GxP Software (Molecular Devices, Sunnyvale, CA, USA). At each dose level of the compounds tested, as well as the untreated (vehicle-only) controls, absorbance values were recorded and compared between different treatment and time points to assess cell growth and viability.

#### 2.15.2. Kinetic Solubility Assay

A stock solution of each active compound in DMSO (10 mg/mL) was prepared and diluted into physiological buffer (pH 7.4) to a concentration of 100 µg/mL, and the mixture was left at room temperature for six hours. After six hours, the sample was centrifuged, and the supernatant was diluted with a methanol-water solution containing an internal standard (IS). The calibration curve was established using seven-point standards prepared by serial dilution in a range that the unknown sample signal would likely fall in and precisely simulating the sample preparation process. The standards and samples were quantified using LC-MS/MS, and unknown concentrations were back-calculated from the calibration curve.

#### 2.15.3. PAMPA Permeability Assay

The parallel artificial membrane permeability assay (PAMPA), an artificial membrane that simulates a biological membrane, was used. A 96-well membrane filter-based microtiter plate system with donor and acceptor compartments separated by the membrane was employed. The compounds were dissolved in 5% DMSO in phosphate-buffered saline (PBS) solution with Lucifer yellow, a dye used to assess membrane integrity. The solution was placed in the donor well, and PBS was added to the acceptor well. The membrane was generated by dissolving lecithin in an inert organic solvent and placed onto a hydrophobic PVDF filter [27]. The plates were then allowed to incubate for 16 h. For each compound, a concentration versus area ratio calibration curve with seven points was plotted, and the concentration of the compounds in the donor and acceptor compartments was quantified using the calibration curve on an HPLC. The apparent permeability (P_e_, cm/s) was calculated using the formula:

(1) $$\mathrm{Apparent}\;\mathrm{Permeability}\;(P_{e})=\left[\frac{V_{D}\times V_{A}}{\left(V_{D}+V_{A}\right)\times Area\times Time}\right]\times \left\{-ln\left[1-\frac{C_{A}(t)}{C_{eq}}\right]\right\}$$

- *V_D_* = donor compartment volume (0.15 mL);
- *V_A_* = acceptor compartment volume (0.3 mL);
- *Area* = area of the membrane (0.3 cm^2^);
- *Time* = time of incubation (57,600 s);
- *C_A_(t)* = concentration of solution in the acceptor chamber after 16 h;
- *C_eq_* = represents the equilibrium concentration;
- Quality control standards, Verapamil (*P_e_* = 16 × 10^−6^ cm s^−1^) for high permeability and theophylline (*P_e_* = 0.12 × 10^−6^ cm s^−1^) for low permeability, were run with each sample set to monitor the consistency of the analysis.

#### 2.15.4. Plasma Protein Binding (PPB)

The rapid equilibrium dialysis (RED) method was used. The compounds were spiked with human plasma at 10 µg/mL concentrations and placed in plasma chambers of the RED device. PBS was placed in the buffer chambers, and the RED device was sealed and allowed to shake in an orbital shaker for four hours at 37 °C to achieve equilibrium. Equal volumes of plasma were added to the aliquots of the buffer chambers and vice versa to create identical matrices. The compounds were then precipitated using methanol (four times the aqueous phase) and centrifuged, and then the compound concentration in the supernatant was quantified using LC-MS-MS. The % free and bound drug concentrations were calculated using Equations (2) and (3), respectively.

(2). % Free drug concentration

(2) $$\%\;Free=\frac{\left[Buffer\;Chamber\;Compound\;Concentration\right]}{\left[Plasma\;Chamber\;Compound\;Concentration\right]}\times 100$$

(3). % Bound drug concentration

(3) % Bound=100−% Free

#### 2.15.5. Metabolic Stability

The metabolic stability of the prodrug was assessed in the mouse liver S9 fraction. Briefly, 5 μg/mL of prodrug was incubated with 1 mg/mL mouse liver S9 fraction supplemented with 1 mg/mL nicotinamide adenine dinucleotide phosphate (NADPH) in Dulbecco’s phosphate-buffered saline at 37 °C. At designated time points (i.e., 0, 0.5, 1, 2 h), 100 μL samples were collected and quenched immediately with 300 μL methanol containing 1 μg/mL internal standard. Samples were vortexed for 30 s and centrifuged at 15,000 rpm for 10 min. Supernatant was assessed by LC/MS.

### 2.16. Data Analysis and Statistics

Cell numbers in mouse cochleae or fish, as well as fish neuromast morphologies, were evaluated in 25 zebrafish per drug and treatment group across three experimental runs. For mouse analyses, 18 cochleae (from 9 mice) per treatment and three experimental repetitions were examined. Images were analyzed at 200X magnification, obtained from different neuromasts and cochlear regions (apex, middle, and base). HCs were quantified in the mouse OC by counting M7a-positive cells, with HC identity further confirmed by phalloidin-labeled stereocilia. Apoptosis and cell proliferation events, on the other hand, were quantified by counting TUNEL- and EdU-positive cells, respectively. Counts were obtained from three randomly selected microscopic fields for each cochlear region, covering at least 1200 μm in total. The number of HCs (IHCs and OHCs) is expressed as units per 200 μm. Cell counting was performed in a double-blind and unbiased manner. A laboratory member conducted the experiments, while another person, who was unaware of the treatment groups to which the cochlea belonged, performed the cell counts. IHCs and OHCs were counted on reconstructed confocal stacks using NIH ImageJ v1.8.0 software. The presence of an HC was defined as an intact, spherical nucleus located in the basal half of an M7a-positive cell. The same person who performed the initial counts recounted a select group of cochleae to determine an intra-rater reliability measure. We used intra-class correlations (ICC) to assess the reliability of the data sets. Reliability statistics were calculated in SPSS v22.0. We used an intra-class correlation coefficient of ≥0.7 to measure strong absolute agreement between the two sets of HC counts being compared. Statistical comparisons between animal groups and treatments were performed using Two-Way ANOVA or Kruskal–Wallis test with post hoc Bonferroni correction for multiple comparisons. Values are presented as ± standard deviation (SD) of the mean. Fluorescence incorporation following treatment with FM1-43 was quantified according to [19] using ImageJ. An unpaired T-test was used to compare the means between treatment groups. *p* < 0.05 was considered significant. Qx treatments were compared to vehicle-only (control) animals. Vehicle-treated animals showed no differences in the number of HCs, neuromast scores, TUNEL, or EdU, and were pooled together as a single control group.

## 3. Results

### 3.1. Synthesis of Qx-294 and 301

**Figure 2:**
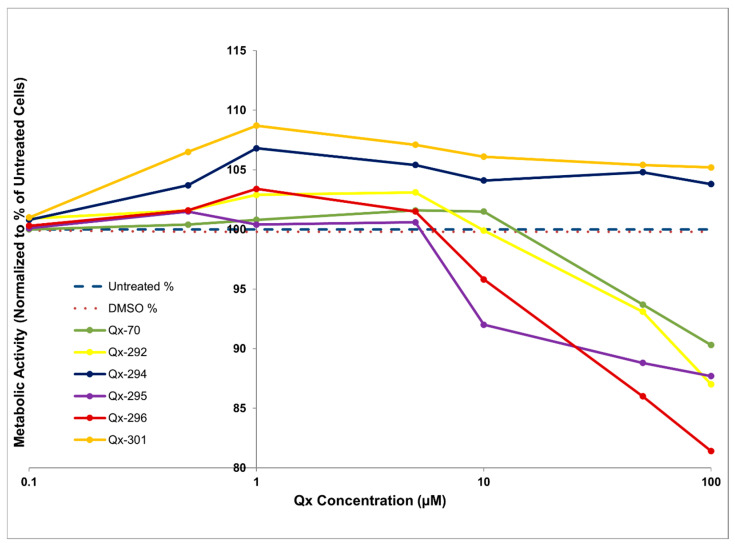
HEI-OC1 Cells metabolic activity vs. different concentrations of Qx analogs measured by a tetrazolium (TACS^®^ MTT) assay. Cells were exposed to Qx analogs (Qx-292, Qx-294, Qx-295, Qx-296, and Qx-301) at a concentration of 0.1–100 μM. Qx-70 was added as an internal control. Metabolic activity was expressed as a percentage of the untreated cells after blank (only reagents without any cells) subtraction and plate-level vehicle normalization (ratio of means). The x-axis shows drug concentration (μM, log scale 0.1–100); the y-axis shows percentage of activity. The dashed horizontal line marks the untreated reference (100%). The dotted line shows the DMSO vehicle across doses. Solid lines with markers indicate responses to Qx analogs. Higher values reflect increased tetrazolium reduction relative to the untreated cells.

To investigate the influence of the nature of substituents (R) on Qx biological activity, we generated a series of 70 quinoxaline derivatives by the addition of various R groups ranging from hydrophilic to lipophilic. Within the 70 different analogs generated, only one chemotype (Qx-70) showed potential for further preclinical optimization (Figure 1A; dotted box). Ten alkylated Qx analogs (Qx-292–Qx-301) were synthesized and isolated (Figure 1B). Briefly, 2-chloroquinoxaline was reacted with substituted amines in the presence of potassium carbonate, yielding final products in moderate yields (7.8–18.7%). All analogs were initially evaluated against the HEI-OC1 cell line to determine their effect on cell proliferation and cytotoxicity. Five of these compounds [Qx-292 (ethyl), Qx-294 (morpholine), Qx-295 (thiomorpholine-substituted), Qx-296 (4-acetylpiperazine), and Qx-301 (pyrrolidine)] demonstrated significant and reproducible proliferative effects greater than those of Qx-70 and the untreated cells (Figure 2; Appendix A). While the TACS MTT assay primarily measures metabolic activity, this activity is generally correlated with cell numbers. As such, we measured the dose–response of metabolic activity in HEI-OC1, Neuro-2A, and HT-1080 cells treated with different concentrations of all five Qx analogs to assess whether the treatment caused the cells to proliferate faster than the control (untreated cells) group during the 24 h incubation period (Figure 2; Appendix A). Consistent with their impact on cell proliferation, Qx-70, Qx-292, Qx-295, and Qx-296 exhibited a high proliferative effect and increased metabolic activity at concentrations ranging from 0.1 μM to 5 μM. However, across all three lines cells’ metabolic and proliferative activities declined at concentrations higher than 5 μM (Figure 2; Appendix A), consistent with a threshold-dependent shift from proliferation and survival (low-exposure dose) to cytostatic (≥5–10 μM) and possibly cytotoxic (≥10–100 mM) for four of the six compounds.

On the other hand, cells treated with different concentrations of Qx-294 and 301 exhibited elevated yet stable metabolic activity (Figure 2; Appendix A) throughout all concentrations and cell lines, informing our decision to focus on these two compounds and on the inner ear-specific (HEI-OC1) cell line for all subsequent experiments.

### 3.2. In Vitro ADMETox

The ADMETox evaluation of QX-294 and QX-301 against HEI-OC1 cells is summarized in Table 2. Qx-294 and Qx-301 had low aqueous solubility, with Qx-294 achieving the highest solubility at approximately 551 ng/mL (Table 2). Despite the highly weak acidic nature of the conjugated 7-amino group, the extensive conjugation of the pi electron system extended from the 7-amino group is likely the primary driver for the poor aqueous solubility by creating strong crystal packing energy. The introduction of ionizable amines will be the focus of the second generation of Qx analogs. In early-stage drug development, PAMPA permeability above 0.5 × 10^−6^ cm/s is considered optimal [27], and both analogs were able to achieve this threshold, suggesting effective membrane permeability and distribution. However, Qx-294 exhibits outstanding permeability at approximately 3.5 × 10^−6^ cm/s. Likewise, Qx-294 is highly protein-bound in plasma, while Qx-301 is approximately 32% bound. Classically, only the free/unbound drug is free to reach its biological target to elicit its pharmacological action; therefore, Qx-301 has strong potential to stimulate significant in vivo efficacy at lower doses compared to Qx-294. This is particularly relevant because, while plasma protein binding rarely leads to drug failure in early development, it remains an essential factor to evaluate because highly protein-bound drugs can cause significant drug–drug interactions. Even though high plasma protein binding can be desirable, as it often leads to improved metabolic stability and a longer drug half-life, lowering protein binding increases the drug’s free fraction, which is needed for effective pharmacodynamics. Some medicinal chemistry strategies to reduce plasma protein binding include increasing polarity or charge. Our lead Qx analogs are weakly basic and have high in vivo efficacy; therefore, increasing basicity to produce more polar or even charged (cationic) compounds should maintain efficacy while decreasing plasma protein binding. Additionally, elevating bulk through pegylated nanoparticle formulations with sustained drug release can also prevent the drug from significantly binding to plasma proteins. Finally, both Qx analogs were determined to be highly metabolically stable against the human S9 fraction, with Qx-294 being exceptionally stable, having a half-life of approximately 57 h.

### 3.3. Increasing Concentrations of Qx-294 and Qx-301 on Zebrafish Neuromasts Do Not Affect the Integrity or Survival of Neuromast HCs In Vivo

**Figure 3:**
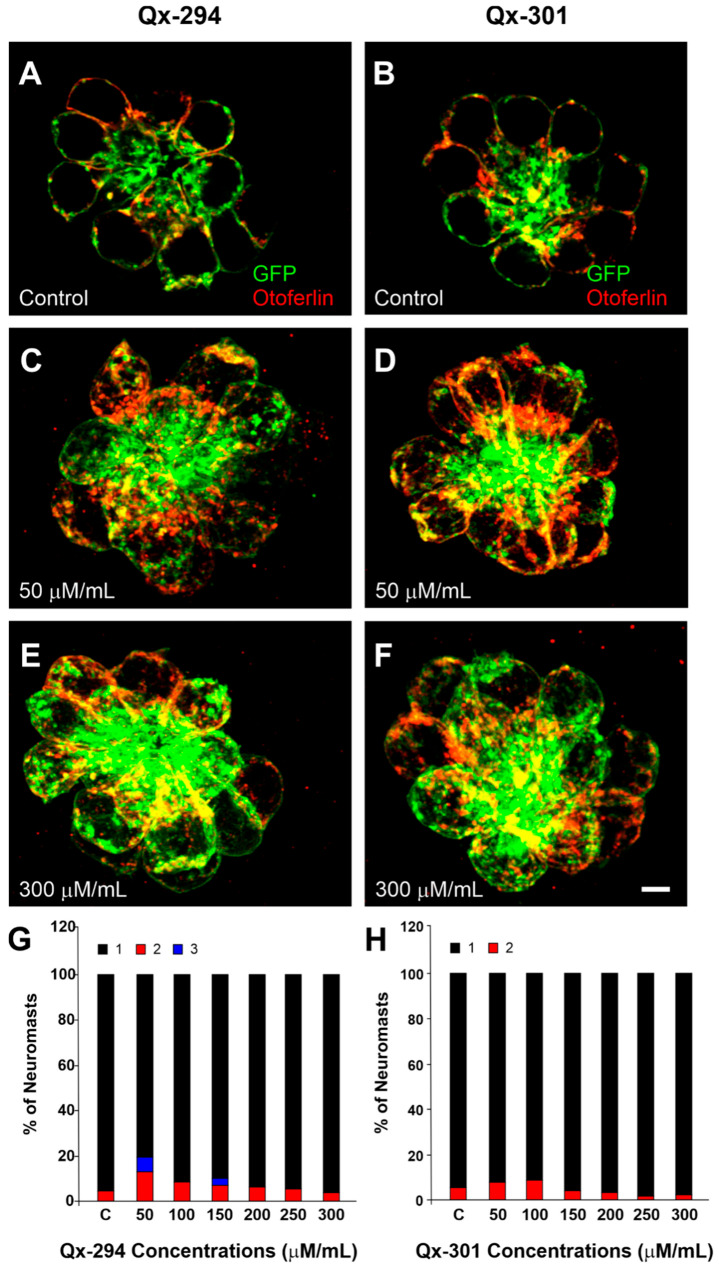
Increasing concentrations of Qx-294 and Qx-301 do not affect the integrity or survival of neuromast HCs in vivo. (**A**–**F**) Regardless of the drug concentration tested, no structural changes were observed in HCs’ structure or neuromast anatomy. Given the lack of variability in the overall integrity of the HCs and neuromast structure, only images for the lowest and highest concentration treatments are shown. (**G**,**H**) An arbitrary scoring protocol was used to rank the morphology of each neuromast after treatment (See Material and Methods for the full score descriptions): 1. normal rosette-like shape, HCs look normal, HC bundles are properly arranged, 2. normal rosette-like shape, a few HCs appear to be missing, but HC bundles are properly arranged, 3. normal rosette-like morphology, but several HCs are gone and HC bundles look disturbed. Except for a few HC losses in Qx-294-treated neuromasts, which were not statistically different from the control samples, no changes to the overall integrity of the neuromasts and HCs were observed. C = Control; values in (**G**,**H**) correspond to μM/mL. In (**A**–**F**), Green = GFP and Red = Otoferlin. Bar = 6 μm.

To identify the optimal dose for proliferation and assess potential in vivo toxicity of Qx-294 and Qx-301 in HCs, 5-dpf zebrafish were treated with increasing concentrations of Qx-294 and Qx-301 (50, 100, 150, 250, and 300 μg/mL) in swimming (E3) medium for 24 h. To examine the effect of the different drug concentrations on the neuromast morphology and HCs’ numbers and integrity, we performed immunofluorescence using otoferlin and GFP antibodies (Figure 3A–F). An arbitrary scoring protocol (see Materials and Methods for the explanation of score assignments) was used to rank the morphology of each neuromast after treatment. Overall, scores for the morphology of neuromasts treated with Qx-294 or Qx-301 at different concentrations did not differ significantly from those of the control group (Kruskal–Wallis test with Bonferroni correction; *p* > 0.05) (Figure 3G,H).

**Figure 4:**
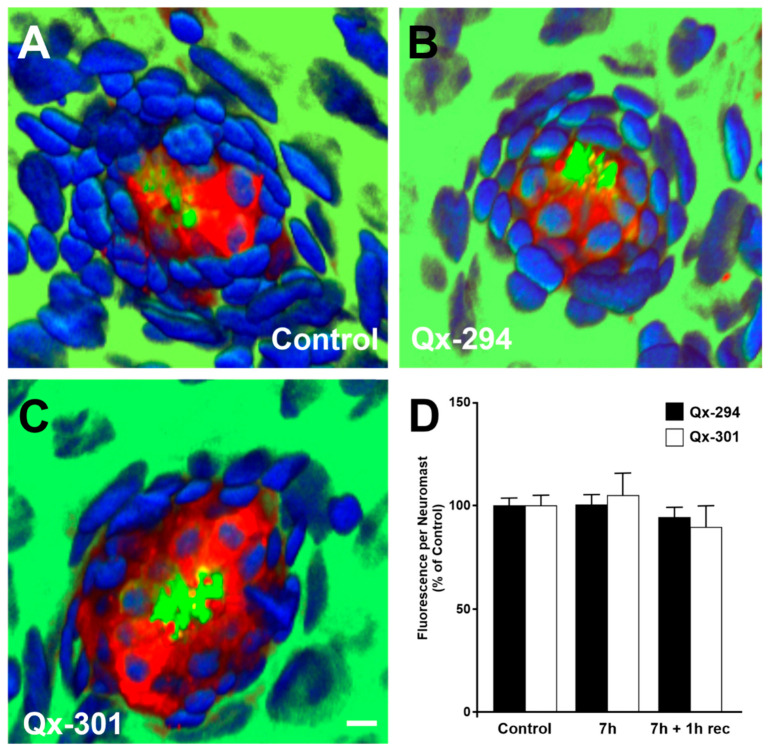
Treatment with Qx-294 or Qx-301 does not abolish mechanotransduction channel activity. 5dpf wild-type larvae were incubated in E3 media alone (**A**) or containing Qx-294 (**B**) or Qx-301 (**C**) at 300 μM/mL for 7 h and immediately assayed for FM1-43 uptake. Alternatively, following incubation with compounds, animals were allowed to recover for 1 h and then treated with the FM1-43 alone (7 h + 1 h recovery). For all groups, neuromasts showed robust dye uptake (Red), indicating open mechanotransduction (MET) channels in both control (**A**) and Qx-treated (**B**,**C**) neuromasts. Phalloidin labels (Green), which label F-actin-rich stereocilia bundles and the cuticular plates of the HCs, confirmed intact bundle morphology. HCs and SCs nuclei are labeled DAPI (Blue). FM1-43 mean fluorescence intensity per neuromast was normalized to the control group (mean ± SEM; n = 25 animals per treatment). Quantification of the fluorescent intensity per neuromast (**D**) was expressed as a percentage of controls. No significant differences were observed between control and any of the treatment groups (unpaired Student’s T-test). Green = Phalloidin, Red = FM1-43; Blue = DAPI; Bar = 6 μm.

While not statistically significant, some differences were observed in neuromast morphology between compounds. For example, although at most concentrations the scores of Qx-294-treated neuromasts were comparable to the control values, at two different concentrations (50 and 150 μM/mL), some HC loss was observed (Figure 3G). On the other hand, at the highest concentrations, Qx-301-treated neuromasts appeared to have an improved morphology than the control samples (Figure 3H). Mechanotransduction is essential for the development, function, and survival of neuromast HCs [28]. FM1-43 served as a proxy for mechanotransduction channel activity in zebrafish treated with Qx-294 and Qx-301 (Figure 4A–C). Consistent with no changes in their mechanotransduction activity, rapid dye entry into HCs was observed in both the control, untreated, and Qx-treated groups for both compounds (Figure 4B,C). Likewise, no visible differences were observed in FM1-43 uptake in fish transferred to fresh media before treatment with FM1-43 (7 h + 1 h recovery). Quantification of the fluorescence intensity in the neuromasts confirmed the visual observations, revealing no significant differences between treatment groups with or without recovery and the control groups (Figure 4D).

### 3.4. Qx-294 and Qx-301 Treatments Lead to Unscheduled Proliferation and Supernumerary Cells on Zebrafish Neuromasts and Mouse Cochlear Explants

**Figure 5:**
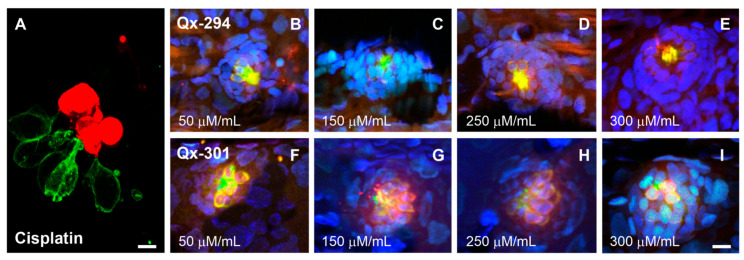
Qx-294 and Qx-301 do not have a cytotoxic effect on neuromast cells. TUNEL assay (red) was performed on zebrafish incubated with vehicle (negative control), 400 μM cisplatin (positive control), or various concentrations of Qx-294 and Qx-301. (**A**) Consistent with its known cytotoxic effects, extensive cell death was observed in cisplatin-treated animals. (**B**–**I**) Regardless of the drug or concentration, no differences in TUNEL results were seen in the experimental animals or between them and the negative control. Animals were also immunolabeled for GFP (green). Due to the lack of variation in the results, only four of the six concentrations tested are shown for each compound. Green = GFP; Red = TUNEL; Blue = DAPI. Bar (**A**) = 6 µm; Bar (**B**–**I**) = 10 µm.

To further investigate the effect of Qx-294 and Qx-301 exposure on zebrafish HC survival, we performed a TUNEL assay. Some of our previous experiments have shown that when the TUNEL reaction or earlier permeabilization steps (e.g., Proteinase K treatment) are too prolonged, it results in tissue structural disruption, leading to the loss of apoptotic nuclei and TUNEL-negative HCs, even when apoptosis has occurred [19]. To eliminate the possibility of neuromast disruption and rule out any possible false negatives, animals were incubated with Qx-294 and Qx-300 for 4, 6, 12, and 24 h. Notably, incubations lasting longer than 12 h were characterized by some neuromast disruption, indicating that the incubations had exceeded the recommended duration. On the other hand, no differences were observed between the shorter incubation periods. To maintain uniform incubation times, all subsequent experiments followed a 6 h incubation period. Positive controls consisted of zebrafish incubated with 400 µM cisplatin for 6 h. Compared to the positive control (Figure 5A), no signs of apoptosis were observed in the neuromasts of zebrafish treated with Qx-294 (Figure 5B–E) or Qx-301 (Figure 5F–I) at any concentration tested.

**Figure 6:**
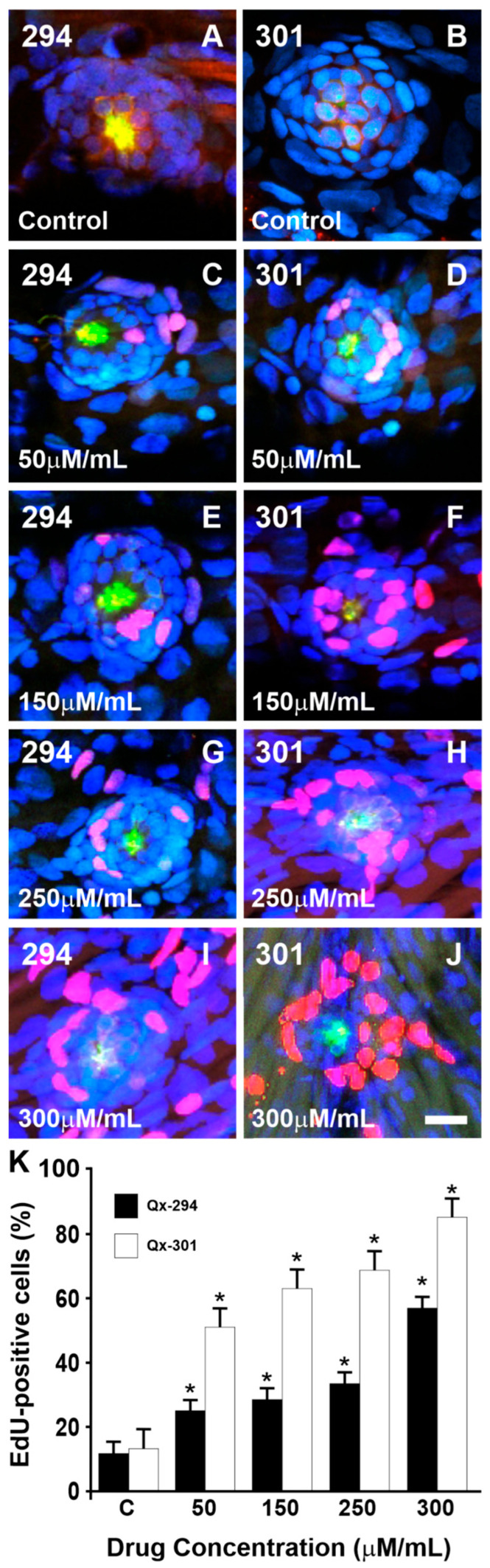
Treatment with Qx-294 or Qx-301 promotes cell proliferation. (**A**,**B**) EdU-labeling (red) was observed in the neuromast cells of control animals, but at a much lower percentage than in the experimental group animals. (**C**–**J**) EdU-positive nuclei were seen in the SCs surrounding HCs following treatment with either Qx-294 or Qx-301. (**K**) The percentage of EdU-positive SCs per neuromast was calculated for each treatment and represented as the mean ± SD. Two-Way ANOVA with Bonferroni correction, * = *p* < 0.05. C = Control group. Green = Phalloidin; Red = EdU; Blue = DAPI. Bar = 10 µm.

Next, we assessed the proliferative effect of Qx-294 and Qx-301 on zebrafish neuromasts (Figure 6A–J). Consistent with their potential impact on HC regeneration, 24 h post-treatment, EdU-positive SCs were observed in neuromasts of fish treated with either Qx-294 or Qx-301 at all concentrations tested (i.e., 50, 100, 150, 250, and 300 μg/mL) (Figure 6C–J). For each treatment, 25 animals were used, and the experiments were repeated three times. Differences between compounds and between concentrations within each compound were tested by Two-Way ANOVA. Three neuromasts per fish were assessed. Notably, HCs were consistently EdU-negative, regardless of the compound or treatment concentration (Figure 6C–J). The number of proliferating cells per compound and concentration was assessed by counting EdU-positive cells in confocal stacks of imaged neuromasts (Figure 6K). Qx-294 and Qx-301-treated fish displayed a significantly higher number of proliferating cells than the control groups (*p* < 0.05; Figure 6K). However, when the results were compared between the two compounds, the number of EdU-positive SCs was significantly higher for Qx-301- than for Qx-294-treated neuromasts (Two-Way ANOVA with Bonferroni correction; *p* < 0.05).

### 3.5. The Effect of Qx-294 and Qx-301 Treatments in the Mouse Cochlea

**Figure 7:**
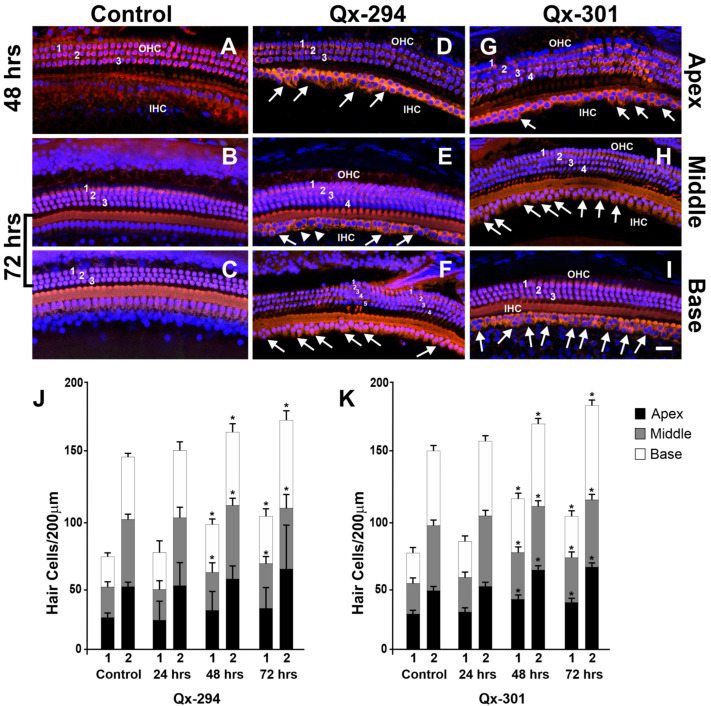
Treatment with Qx-294 or Qx-301 leads to supernumerary cells in the mouse OC. (**A**–**C**) Control cochlea (vehicle-only). (**D**–**F**) Cochleae treated with Qx-294. (**G**–**I**) Cochleae treated with 301. Overall, the sensory epithelium architecture was preserved in treated ears, but supernumerary M7a-positive HCs were observed in both inner and outer HC domains after drug exposure (**D**–**I**) vs. (**A**–**C**). At 24 h, total HC counts did not differ from those of the controls (**J**,**K**). By 48–72 h, quantification showed significant increases in both OHC and IHC numbers in compound-treated mice (*p* < 0.05; **J**,**K**). In the apex, Qx-294 showed a variable, non-significant trend towards more HCs (**D**,**J**), whereas Qx-301 produced a consistent, significant increase in apical HCs (**G**,**K**). In both compound treatments, supernumerary cells aligned along the cochlear axis to form additional rows, disrupting the typical 3:1 OHC:IHC pattern (**D**–**I**). Data are mean ± SD of total M7a-positive cells per sample. Arrows = supernumerary IHCs; arrowheads (**E**) = enlarged cytoplasm/nuclei consistent with cells in G1 phase. Red = M7a; Blue = DAPI. * = *p* < 0.05. Bar = 10 µm. Numbers 1–5 on (**A**–**I**) represent the number of OHC rows. On (**J**,**K**), 1 = IHC; 2 = OHC.

**Figure 8:**
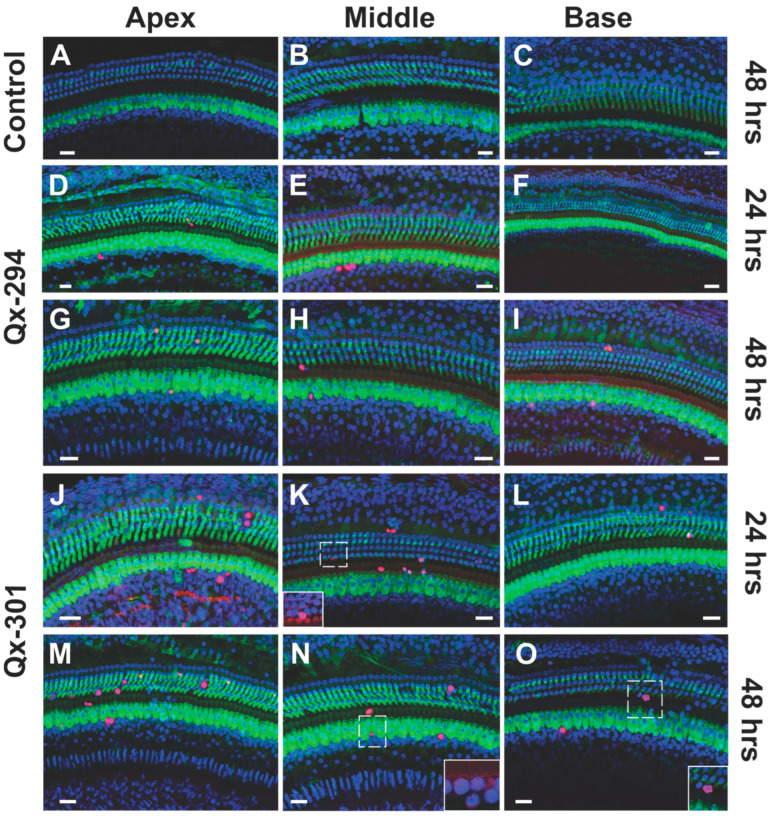
Cell proliferation in the mouse OC following Qx-294 or Qx-301 treatment. (**A**–**C**) No EdU labeling (red) was seen in the OC of control animals, regardless of the treatment time (data shown only for a 48 h sample). (**D**–**O**) In contrast, EdU-positive cells were observed in the outer and inner HCs (green) regions at 24 and 48 h intervals, but not at 72 h post-treatment. Highlight boxes in (**K**,**N**,**O**) display close-up details of cell nuclei at various stages of proliferation. As demonstrated by triple-channel (M7a/EdU/DAPI), EdU-positive nuclei were consistently observed adjacent to, but not colocalizing with, M7a-positive outer or IHCs, suggesting that the proliferative activity induced by Qx-294 or Qx-301 treatment occurred within the SCs and does not represent direct hair-cell regeneration. Green = M7a; Red = EdU; Blue = DAPI; Bar = 10 µm.

**Figure 9:**
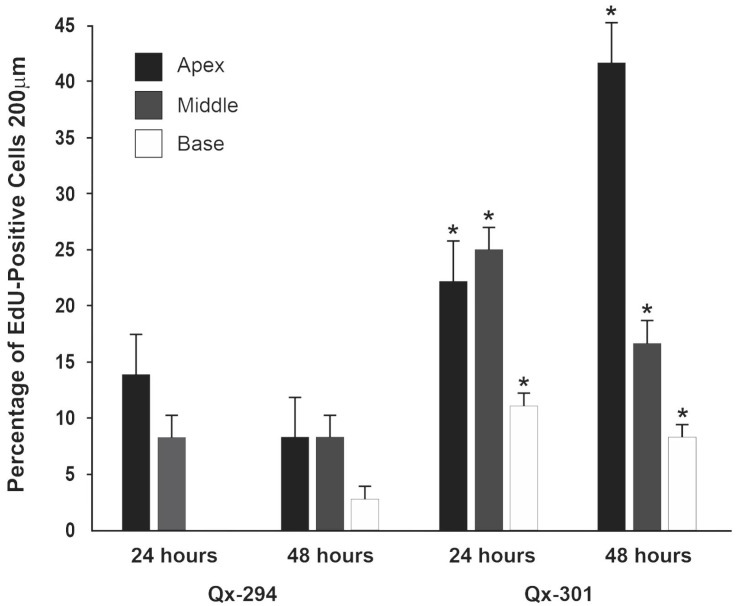
Quantification of EdU-positive nuclei following Qx-294 or Qx-301 treatment. The total number of EdU-positive cells per sample was quantified for each treatment and represented as mean ± SD. These data represent averaged values, which may differ from the appearance of individual representative images shown in Figure 8. At the 24 h treatment, both apical and middle turns showed a significant increase in EdU labeling relative to controls, but not between themselves, resulting in comparable mean percentages. However, at 48 h, the percentage of EdU-positive cells in the apical turn was significantly higher than in the control or the middle turn. Comparisons between treatments were performed using One-Way ANOVA with post hoc Bonferroni correction for multiple comparisons. EdU-positive nuclei consistently appeared adjacent to the Outer and IHCs, with no EdU+/M7a+ colocalization observed. * = *p* < 0.05.

Next, we assessed the effect of Qx-294 and Qx-301 treatment on the mouse cochlear anatomy. Male and female mice were assigned to different drug treatment groups (Qx-294, Qx-301, vehicle-control) and time of post-treatment evaluation (24, 48, or 72 h). All animals were P28 at completion of Qx treatment. After each time, animals were perfused and fixed overnight in 4% paraformaldehyde (PFA) at 4 °C. Immunohistochemistry experiments were performed using M7a antibody and DAPI counterstaining (Figure 7A–I). Supernumerary M7a-positive cells were observed in the OC of mice treated with either drug after all three treatment times (Figure 7J,K). At 24 h post-treatment, only a few supernumerary M7a-positive cells were observed, and the difference was not statistically significant from that of the control animals (Figure 7J,K). Significant differences in the number of supernumerary cells per treatment and some regions of the sensory epithelia were observed in the 48 and 72 h post-treatment groups (Figure 7A–K). Overall, the supernumerary OHCs were arranged following the cochlear axis and forming supernumerary rows of M7a-positive OHCs, in the lesser epithelial ridge (LER) area (Figure 7C–H). On the other hand, supernumerary M7a-positive cells in the IHCs’ regions were organized in clusters near the regular row of IHCs, where the inner phalangeal (IPh) and inner border (IB) cells usually are found (Figure 7B–I). Although the progenitor cells that lead to the origin of the supernumerary cells remain to be identified, a few enlarged M7a-positive cells, characteristic of Gap (G) 1 phase cells, were observed on Qx-294-treated cochleae in the 48 h treatment group (arrowheads in Figure 7E). That observation, combined with the physical location of those supernumerary cells, supports the hypothesis of a mitotic origin from IB and IPh cells, which have been shown to possess some latent mitotic competence [29,30]. To further explore that hypothesis, we assessed the proliferative potential of both compounds in the mouse OC (Figure 8A–O). To that end, at completion of treatment (i.e., 24, 48, 72 h), mice were injected with the thymidine analog EdU (50 mg/kg) and killed 4 h after injection. All animals were P28 at the completion of the drug treatments. Although the density of EdU-positive cells was lower than that observed in zebrafish experiments, a similar pattern was observed in mice treated with either compound (Figure 8D–O), with a higher percentage of EdU-positive cells at both 24 h and 48 h treatment times (Figure 9; unpaired T-test, *p* < 0.05). Notably, while supernumerary HCs were observed in samples from the 72 h treatment group (Figure 7K,J), no proliferating (EdU-positive) cells were observed at that time point, suggesting that the peak of proliferation may have happened earlier between 24–48 h post-treatment. Notably, no proliferating cells were observed in the OC of control mice. It is important to highlight that, similar to zebrafish neuromasts, EdU-positive nuclei were consistently observed adjacent to, but not colocalizing with, M7a-positive inner or OHCs. Although triple-channel imaging (M7a/EdU/DAPI; Figure 8) supports the premise that all EdU-positive cells were located within the SC domain of the OC, no EdU+/M7a+ cells were detected in this study, indicating that the proliferative activity induced by Qx-294 or Qx-301 treatment occurred within SCs and does not represent direct HC regeneration. To complete our analysis, we sought to examine the effect of Qx-294 and Qx-301 treatment on the survival of supernumerary and regular sensory cells, we performed a terminal deoxynucleotidyl transferase dUTP nick end labeling (TUNEL) assay. Consistent with previous results in zebrafish, no signs of apoptosis were observed in the OC of mice treated with Qx-294 or Qx-301 in any treatment group.

### 3.6. Assessing Auditory Function in Qx-294- and Qx-301-Treated Mouse Cochlea

**Figure 10:**
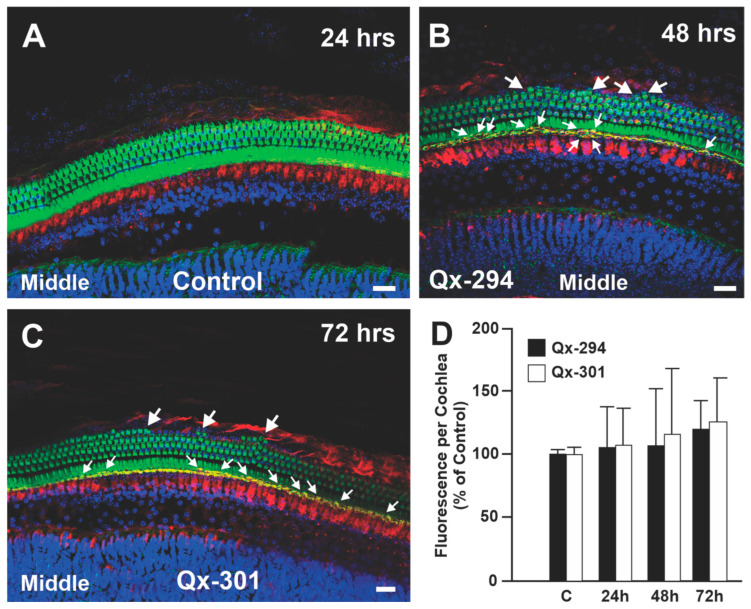
Treatment with Qx-294 or Qx-301 does not abolish mechanotransduction channel activity in the mouse OC. (**A**–**C**) FM1-43 uptake in control (**A**), Qx-294 (**B**), and Qx-301 (**C**) mouse cochlear HCs. FM1-43 uptake was observed in both OHC and IHC of the control and treatment groups. (**D**). Quantification of the fluorescence intensity per cochlea was expressed as a percentage of the controls. Since no differences were observed in FM1-43 uptake throughout the length of the OC, only the middle turn is shown for each group and time point. No significant differences were observed between the control and treatment groups at any experimental time point (One-Way ANOVA with post hoc Bonferroni correction for multiple comparisons). Arrows = supernumerary HCs. Green = Phalloidin; Red = M7a; Blue = DAPI. Bar 10 µm.

Mechanotransduction channels are central to the activity of sensory HCs and are essential for hearing function [28]. Similarly to the zebrafish experiments, FM1-43 provided indirect functional information on the viability of the regular and supernumerary sensory cells treated with QX-294 or QX-301 (Figure 10A–C). Despite the length of treatment with either compound, microscopic examination of the FM1-43/phalloidin immunohistochemical preparations revealed no differences between experimental and control samples, supporting that rapid dye entry into the HCs was comparable between treatments. (Figure 10D). Quantification of the fluorescence intensity incorporated by the cochleae did not show any significant differences from the control samples (Figure 10D), indicating that neither Qx-294 nor Qx-301 blocks the mechanotransduction channels and supporting the premise that both regular and supernumerary HCs are functional. Of note, while not statistically significant, some variability in the fluorescence intensity between compounds and treatment length may be attributed to the presence of supernumerary HCs in the cochleae of animals treated with either compound (Figure 10B,C, arrows).

**Figure 11:**
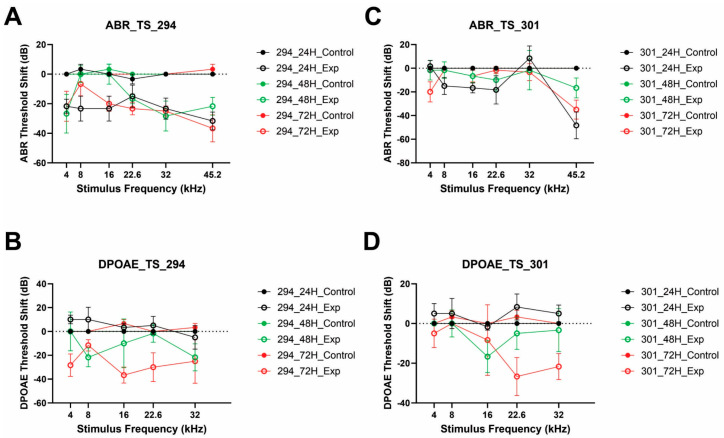
Mice treated with Qx-294 and Qx-301 displayed improved hearing compared to control animals. (**A**-**D**) Audiometric threshold shift (dB) plotted as a function of frequency (kHz) for mice treated with Qx-294 (**A**,**B**) and Qx-301 (**C**,**D**). Controls consisted of vehicle-only treated animals. Threshold shift = pre-treatment–post-treatment. Compared to the control groups, animals treated with either compound showed a modest improvement in hearing threshold beginning at 16 kHz (Qx-294) and 22.6 kHz (Qx-301). The biological significance of this improvement remains to be determined, and longer-term assays will be required to assess whether this early change progresses into more robust or broad functional gain. “0” on the ordinate represents the baseline ABR/DPOAE thresholds for the control animals.

Functional evaluation of the auditory functions after treatment with each of the compounds [i.e., auditory brainstem responses (ABR) and distortion product otoacoustic emissions (DPOAE)] were carried out 24 h before and at 24, 48, and 72 h after drug treatment (Figure 11A–D). ABR and DPOAE threshold shifts were calculated from the three treatment groups (24, 48, and 72 h) post-treatment with either Qx-294 (Figure 11A,B) or Qx-301 (Figure 11C,D) in adult male and female C57BL/6J mice (P24–30; N = 9 animals per treatment or control groups). The control group consisted of animals treated only with the vehicle. Compared to the control groups, mice treated with Qx-294 (Figure 11A) or Qx-301 (Figure 11B) showed improved hearing for all test frequencies, with greater benefits at the higher frequencies and more extended waiting periods following drug treatment (i.e., 48 and 72 h) (Two-Way ANOVA; *p* < 0.01). Moreover, analyses revealed no significant differences in auditory function or treatment duration between the two compounds, supporting the premise that neither Qx-294 nor Qx-301 harms auditory function and further confirming their safety at the tested doses. Similarly, DPOAE amplitudes and thresholds improved, particularly at higher frequencies, where the treatment thresholds were significantly lower than those of the control group (Figure 11C,D) (Two-Way ANOVA; *p* < 0.01). A comparison between the three treatment groups per frequency showed a modest, yet consistent threshold improvement starting at 16 kHz for mice evaluated at 48 and 72 h post-treatment (Figure 11C,D) and found to be statistically significant for the 72 h group at all frequencies above 16 kHz (Qx-294) and above 22.6 (Qx-301) (Figure 11C,D) (Two-Way ANOVA; *p* < 0.05), suggesting an early, localized functional effect of treatment. Consistent with our histological and immunocytochemical assessments, no statistically significant differences were observed between the 24 h treatment and control groups for both ABR and DPOAE assessments (Figure 11A–D).

## 4. Discussion

Genetic and pharmacological manipulation aimed at HC regeneration has driven the shift from basic science to potential human treatments for hearing loss. However, treating hearing loss through HC regeneration is still in the early to mid-stages. Quinoxalines are heterocyclic nitrogen-containing compounds with known antioxidant, anti-inflammatory, and anti-apoptotic properties [19,20,21]. Previous work by our group has underscored the potential of quinoxaline (Qx) in HC protection from ototoxic drugs [19]. Utilizing classic rational drug design approaches, we systematically functionalized the 2-position on quinoxaline with substituted amino groups with straight-chain and cyclic analogs. We have expanded those findings by generating Qx chemotypes and testing their effect in vitro and in vivo. Ring-constrained analogs (highlighted by our two leads Qx-294 and Qx-301) showed more promising potential over straight chain. However, both hydrophilic (Qx-294, morpholine) and lipophilic analogs (Qx-301, pyrrolidine) demonstrated high translational potential, enabling further development to optimize their ADME profiles. Overall, our findings suggest that both Qx-294 and Qx-301 have the potential to modulate proliferative pathways in the zebrafish and mouse sensory epithelia, a process crucial for tissue maintenance and regenerative potential in the mammalian inner ear.

SCs in the zebrafish neuromasts play a dual role: providing structural support for mechanosensory HCs and serving as progenitors during HC regeneration [2,4,5,7,10,15,16,17,18,31]. Our results indicate that Qx-294 and Qx-301 can promote SC proliferation even in the absence of overt injury, implying activation of cell cycle entry under homeostatic conditions. This is particularly notable, as most documented proliferation in zebrafish neuromasts and neonatal mouse OC occurs following damage-induced signaling, often mediated by Wnt, Notch, or FGF pathways [4,30,31,32,33,34,35,36,37,38,39,40,41,42]. Spontaneous SC or HC proliferation is essentially absent in the postnatal and adult mouse OC. Previous studies by our group and others have shown that forced disruption of the pRBs [15,16,17,43,44] and p27kip1 [45,46] checkpoints can trigger limited cell-cycle reentry, but often at the cost of HC survival and function. The current results raise the possibility that these two compounds bypass or modulate upstream injury-dependent triggers by directly or indirectly activating proliferative cascades. Indeed, evidence already exists showing that Erdafitinib, the first FDA-approved quinoxaline derivative, modulates the MAP3K1-IKK-NFβ-κB signaling pathway, which acts as a central hub for integrating a complex and multifaceted role in cell growth, proliferation, and survival [47,48,49]. In previous work [19], we have demonstrated that Qx-mediated NF-κB activation leads to HC protection from ototoxicity-induced apoptosis. We have also shown that upregulation of Cyclin D1, a cell-cycle driver downstream from NF-κB, is sufficient to push otherwise quiescent HCs and SCs towards proliferation [18].

Drug-induced SC proliferation has important implications for sensory HC regeneration. In zebrafish, where HC replacement is a natural process, enhanced SC division following injury is a prerequisite for robust HC replacement and regeneration [4]. On the other hand, adult mammalian cochlear SCs are largely post-mitotic, and their proliferative quiescence poses a substantial barrier to regeneration [3,8,9,11]. Multiple studies show that mammalian cochlear SCs can be induced to re-enter the cell cycle, proliferate, and generate new mitotically derived HCs under defined genetic or pharmacological conditions [11,15,16,17,18,31,46,50]. In that light, pharmacological agents capable of expanding the SC pool—as suggested by the presence of EdU-positive cells exclusively in areas normally occupied by SCs in both zebrafish and mouse treated with either Qx-294 or Qx-301—could potentially prime the sensory epithelia for SC proliferation following ototoxic insult or mechanical damage. It is also important to note that, because EdU incorporation was confined to the SCs’ region and the observation window in the present study was short (24–72 h), our current findings may represent an early pro-proliferative effect rather than evidence of HC regeneration. Demonstration of proper regeneration will require lineage tracing, longer-term maturation studies, and functional assessments in an injury model, including evaluation of synaptogenesis and reinnervation.

While cell proliferation was observed in animals treated with either compound, the magnitude and distribution of proliferating cells varied between compounds, suggesting that the underlying mechanisms may differ. In this context, we hypothesize that one possible mechanism of action may involve the co-activation of canonical proliferative pathways, such as the Wnt/β-catenin and the MAP3K1–IKK–NF-κB pathways [39,51,52]. The crosstalk between these two pathways and its impact on pro-growth signal amplification is well established in the auditory system and beyond [31,52]. As such, one could predict that Qx-induced activation of the Wnt/β-catenin pathway would prime SCs for cell cycle re-entry and lineage plasticity, while MAP3K1–IKK–NF-κB activation would sustain their survival and enhance proliferation by stabilizing β-catenin and co-inducing CyclinD1/c-Myc [48,49,50,51]. Together, co-activation of these two pathways, along with fate cues such as downregulation of proliferative inhibitory signals like Notch-mediated lateral inhibition—a process demonstrated in Drosophila neuroblast lineage [53,54], during newborn mice cardiomyogenesis, and in the mouse OC following acoustic trauma [55,56]—would create a permissive environment for the mitotic regeneration of auditory HCs. Future transcriptomic or pathway-specific reporter assays will be required to test our hypothesis and elucidate the mechanism(s) leading to cell proliferation and possible differentiation (as assumed from M7a expression in supernumerary cells) as we work to improve these compounds and generate new series. Overall, our present findings expand the repertoire of compounds known to influence the regenerative potential of cochlear SCs and sensory HCs’ replacement, paving the way for further studies to investigate whether the observed proliferation in mouse cochleae and zebrafish neuromasts translates into enhanced HC replacement capacity following the loss of existing HCs.

Although mammalian HCs are unable to regenerate spontaneously, several preclinical studies support the potential of drug-based strategies to trigger limited SC proliferation and HC regeneration with measurable hearing improvement [50,57,58,59]. Induction of HC regeneration with functional recovery has been reported following inhibition of the Notch pathway [42,57], overexpression of *Atoh1* [60,61], and deletion of key cell cycle regulators [15], among others. Similarly, inhibition of γ-secretase with LY411575 following acoustic trauma [57] or adenoviral-mediated *Atoh1* expression in chemically deafened mice led to the generation of new OHCs from existing SCs and partial hearing restoration in the mid-frequency range [60]. While modest and variable, the extent of cell proliferation in Qx-294 and Qx-301-treated mice expands on the existing evidence that mammalian SCs retain latent regenerative potential that can be pharmacologically unmasked and improved. We previously reported on a quinoxaline analog [19] that protects the functional integrity of neuromast HCs in zebrafish exposed to ototoxic drugs. Consistent with that, the present observation that functional HC-like supernumerary cells in Qx-294 and Qx-301-treated mice was accompanied by a robust and quantifiable improvement in ABR threshold (in the mid-to-high frequency range) and functional gain, suggest that sensory HC replacement, survival, and synaptic stability are tightly interconnected processes, as such, thinking about them together may be essential for understanding, designing, and improving strategies for hearing restoration.

The dynamic interaction between cell proliferation and differentiation in the mammalian OC is regulated by a tightly coordinated spatiotemporal program, where the apical prosensory cells are the first to become postmitotic but the last to differentiate [31,46,62]. Consequently, while HCs and SCs in the apex may retain proliferative plasticity longer into postnatal development, proliferative properties decrease from the apical to the basal cells in the basal turn of the cochlea [15,16,17,18,62]. This intrinsic characteristic of the mammalian inner ear, however, creates a therapeutic paradox: apical HCs may be “easier” to regenerate, yet basal turn sensory HCs are more vulnerable to ototoxicity, noise trauma, and loss [63]. Consistent with multiple other reports, our current study demonstrated that the region with the strongest Qx-induced proliferation (Apex) is also the region least in need of replacement in clinical scenarios. Because basal turn HC loss predominates in clinical contexts, Qx-mediated apical-dominant proliferation will have limited functionality unless it can be strategized to increase its access and residency in the basal regions of the cochlea—e.g., round-window or canalostomy delivery approaches combined with slow-release hydrogels, nanoparticles, or viscosity-enhanced vehicles that can steepen a base-favoring gradient.

## 5. Conclusions

This study described the synthesis and in vitro and in vivo evaluation of Qx-base small molecules as promising candidates for pharmacological stimulation of auditory HC regeneration. By expanding the structure–activity landscape of the original Qx scaffold, we identified two analogs, Qx-294 and Qx-301, with optimized medicinal chemistry features, including favorable ADME properties, rapid absorption in zebrafish and mouse models, and the ability to induce cell proliferation in zebrafish neuromasts and mouse OC without triggering apoptosis. Although the exact mechanism behind Qx-mediated proliferative response remains to be elucidated, this report provides a crucial foundation for future work to clarify pathway specificity, determine optimal dosing and delivery methods, and assess long-term safety and functional auditory recovery. Continued refinement of this compound series, along with extended in vivo efficacy studies, will be necessary to fully establish their translational potential. Overall, this work advances pharmacological approaches towards HC regeneration and supports broader efforts to address sensorineural hearing loss through future regenerative therapeutics.

## Figures and Tables

**Table 1:** Experimental Animal Allocation.

Experiment	Animal Model	N Per Compound (Qx-294 and Qx-301)	Total
Immunolabeling	Zebrafish	25	50
	Mice	9	18
EdU	Zebrafish	25	50
	Mice	9	18
TUNEL	Zebrafish	25	50
	Mice	9	18
FM1-43	Zebrafish	25	50
	Mice	9	18
Auditory Function	Zebrafish	-	-
	Mice	9	18

**Table 2:** Structural variations and cytotoxicity of Qx-294 and Qx-301.

Compound Code	Solubility ^a^ (ng/mL)	Permeability ^b^ (×10^−6^ cm/s^−1^)	PPB ^c^ (% Bound)	Metabolic Stability ^d^ t_1/2_ = hour	Cytotoxicity ^e^ (IC_50_, μg/mL) *
Qx-294	551 ± 24	3.5 ± 3.0	88.5 ± 4.0	57.2 ± 4.7	>20
Qx-301	365 ± 28	0.77 ± 0.91	32.3 ± 4.3	9.1 ± 8.4	>20

^a^ Kinetic solubility assay; ^b^ PAMPA permeability assay; ^c^ mice plasma protein binding; ^d^ metabolic stability against human S9 fraction; ^e^ cytotoxicity was determined against HEI-OC1 cells. * The criteria of cytotoxicity, as established by the National Cancer Institute (NCI), is an IC_50_ < 20 μg/mL.

**Figure A1:**
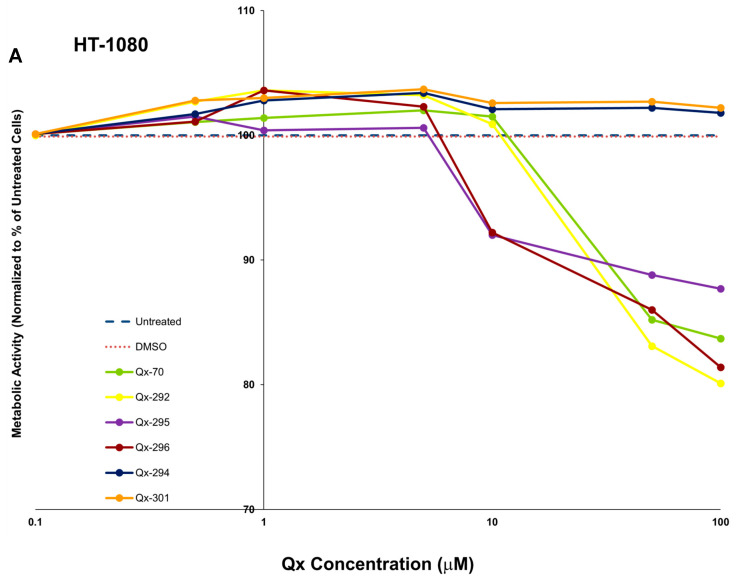

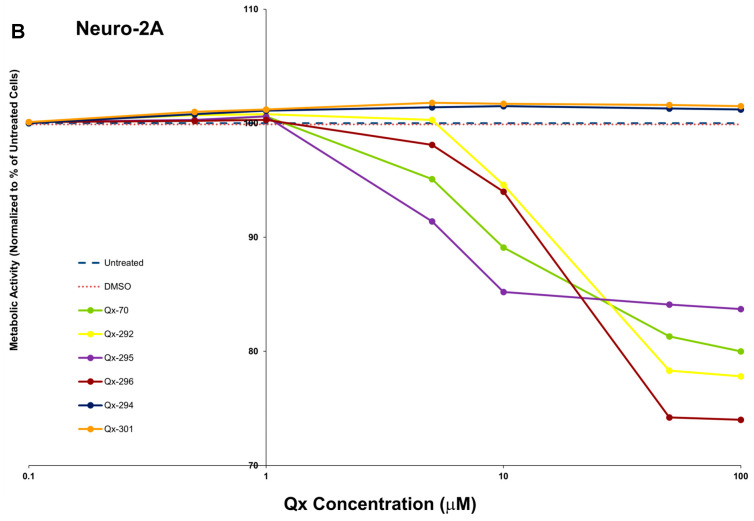
Metabolic Activity Vs. Concentrations of Qx analogs measured by a tetrazolium (TACS^®^ MTT) assay in HT-1080 (**A**) and Neuro-2A (**B**). Cells were exposed to Qx analogs (Qx-70, Qx-292, Qx-294, Qx-295, Qx-296, and Qx-301) at concentrations ranging from 0.1 to 100 μM. Qx-treated HEI-OC1 (Figure 2), HT-1080 (**A**), and Neuro-2A (**B**) cells showed highly similar MTT response profiles. For cells treated with Qx-70, Qx-292, Qx-295, and Qx-296, low-dose exposure (≤5 μM) caused mild increases (≤9%) in absorbance compared to DMSO controls and untreated cells, while higher concentrations (≥5 μM) caused moderate reductions. This indicates a threshold-dependent shift from pro-survival and proliferation to cytostatic (≥5–10 μM) and possibly cytotoxic (≥10–100 μM) effects. In contrast, cells treated with Qx-294 or Qx-301 remained in a modest yet active proliferative state across all tested concentrations. The similar Dose–Response curves among neuronal, mesenchymal, and auditory cell lines suggest that the activity of the Qx derivatives may not be limited to a single cell type but involves a lineage-independent cellular process, possibly a conserved metabolic or stress-response pathway. Prior studies have shown that quinoxaline derivatives regulate NF-kB- and MAPK-linked survival signaling, reduce oxidative stress, and influence mitochondrial redox homeostasis [48,49,50,51]. Therefore, the comparable MTT profiles across the three cell types support the idea that the Qx compounds tested work through a shared cytoprotective mechanism, reinforcing the translational relevance of the findings in HEI-OC1 cells.

## Data Availability

The raw data supporting the conclusions of this article will be made available by the authors on request.

## Abbreviations

The following abbreviations are used in this manuscript:

HC	Hair Cell (s)
SC	Supporting Cell (s)
Qx	Quinoxaline
HEI–OC1	House Ear Institute-Organ of Corti 1
ADME	Absorption, Distribution, Metabolism, & Excretion
ABR	Auditory Brainstem Response
DPOAE	Distortion Product Otoacoustic Emissions
OC	Organ of Corti
TLC	Thin Layer Chromatography
Q1	Single Quadrupole Analyzer
IACUC	Institutional Animal Care and Use Committee
GFP	Green Fluorescent Protein
PFA	Paraformaldehyde
IO4	Infraorbital 4
OP1	Opercular 1
M2	Mandibular 2
O	Otic
MI2	Middle 2
IP	Intraperitoneal
DMSO	Dimethyl Sulfoxide
FFT	Fast Fourier Transform
TDT	Transducers
MTT	3-(4,5-dimethylthiazol-2-yl)-2,5-diphenyltetrazolium bromide
FBS	Fetal Bovine Serum
PAMPA	Parallel Artificial Membrane Permeability Assay
PBS	Phosphate-Buffered Saline
NADPH	Nicotinamide Adenine Dinucleotide Phosphate
IHC	Inner Hair Cell (s)
OHC	Outer Hair Cell (s)
ICC	Intra-Class Correlations
SD	Standard Deviation
IS	Internal Standard
ADMETox	Absorption, Distribution, Metabolism, Excretion, & Toxicity
NCI	National Cancer Institute
MET	Mechanotransduction
LER	Lesser Epithelial Ridge
IPh	Inner Phalangeal
IB	Inner Border Cells
G	Gap
